# Supplementary material for: Grip power test: A new valid and reliable method for assessing muscle power in healthy adolescents
Source: PLoS One. 2021 Oct 20;16(10):e0258720. doi: 10.1371/journal.pone.0258720 (PMC8528297; doi:10.1371/journal.pone.0258720)
Supplement: Attachment — Submitted filename: Reviewer 2.docx [file pone.0258720.s001.docx]

Dear Reviewer 2

We are writing to provide an answer to the suggestions given. The modifications are marked in bold type in the manuscript. Also, in this letter, we present the modifications performed and/or answers to each question.

**Comment 1:** Lines 65-66: A brief paragraph about the importance and the purposes of GripW test) should be added. Please, specify in all paper what is the reliability assessed (inter-day, intra-day)

**Answer:** A short paragraph on the importance of the GripW test was added. In addition, "inter-day reliability" was included.

**Comment 2:** Line 88: Please, add the sample size calculation

**Answer:** In the manuscript, we make it explicit that the sample was for convenience. For this reason, we do not have a sample size calculation.

We relied on validity and reliability studies with fewer than 20 participants (doi 10.1080/17461391.2019.1704068; doi 10.1519/jsc.0000000000003118).

**Comment 3:** Line 90: I suggest checking SD of the stature

**Answer:** Corrected

**Comment 4:** Lines 92-93: I suggest replacing this information in result paragraph

**Answer:** This information was replaced in the results

**Comment 5:** Lines 105-106: Please, specify the warm-up procedures? Were they standardized or individualized?

**Answer:** It was specified that the warm-up was standardized for all participants.

**Comment 6:** Lines 106-108: Was the position choose based on a reference?

**Answer:** Yes, the position was based on references. The reference was included.

**Comment 7:** Lines 111-112: Was the handgrip test performed with both hands? (Please, specify before lines 112) If so, were randomized? Was performed a familiarized test? Comment 1: During the test was the participant verbally encouraged? Should the operator influence the measure?

**Answer:** All comments were specified in the manuscript.

**Comment 8:** Lines 111-112: Have the procedures been taken by some references?

**Answer:** Yes, the reference was included in paragraph.

**Comment 9:** Lines 116-117: Please, specify the warm-up procedures? Were they standardized or individualized?

**Answer:** It was specified that the warm-up was standardized for all participants.

**Comment 10:** Lines 130-131: How the authors verified this “Also, participants were asked not to include shoulder, elbow, and wrist movements in the execution of the test”?

**Answer:** It was specified that a member of the research team carried out the qualitative evaluation of each execution.

**Comment 11:** Lines 150-151: Please, specify the reliability assessment if was intra-day or inter-day. It was not clear also considering the experimental protocol above written. Were the tests conducted at the same time?

**Answer:** The following was corrected throughout the manuscript: "inter-day reliability".

**Comment 12:** Line 154: “are presented as means and standard deviations” should be “are presented as mean and standard deviation”

**Answer:** Corrected

**Comment 13:** Line 155: Please, specify the criteria to validity assess.

**Answer:** It was made explicit that the validity determined was concurrent validity and that "the load-power curve and the respective linear regression equation for all participants" developed in the GripW test were used to determine it.

**Comment 14:** Line 160: Please, report all the classification for CV and ICC

**Answer:** We state that "Acceptable inter-day reliability was determined as a CV < 10% and ICC > 0.85". We believe it is unnecessary to state the entire classification based on this antecedent since the parameters are either met or not met. There is no in-between.

**Comment 15:** Line 162: Please, add a reference for r coefficients

**Answer:** Reference included.

**Comment 16:** Table 1: Please, specify what the authors meant with Load 1, 2, 3,,,,. Moreover, what the authors reported in tables 1 as test and retest? How they explained these higher SDs almost the same of mean?

**Answer:**

Table 1 shows the mean test-retest powers for the right and left hands. This information was also corrected in Table 1.

Only the SDs of the first load, both in test and retest, were more significant than the mean values. Since there was a large dispersion among the 21 participants.

**Comment 17:** Lines 186-188: The sentence in lines 184-186 should test the validity but not the reliability. Please, add a sentence for reliability results.

**Answer:** Sentence included

**Comment 18:** Line 248: Please, specify hot has been determined the muscle mass? To assess the quality of the muscle it is needed other measurements. Moreover, have been determined such as forearm length, hand length? Please, review the entire paragraph.

**Answer:** We added explanations related to muscle quality assessment. In addition, we gave responses to comments made by the reviewer.

**Comment 19:** Lines 257-258: I suggest avoiding data in the discussion part.

**Answer:** Corrected

**Comment 20:** Lines 259-260: Please, review accuracy and reliability terms because in these lines were used as synonymous

**Answer:** The term used was corrected.
